# Supplementary material for: Trait anxiety and depressive rumination mediate the effect of perceived childhood rearing on adulthood presenteeism
Source: PLoS One. 2023 Aug 3;18(8):e0289559. doi: 10.1371/journal.pone.0289559 (PMC10399792; doi:10.1371/journal.pone.0289559)
Supplement: S1 Appendix — (PDF) [file pone.0289559.s001.pdf]

S1 Appendix. Supplementary figures

(A) Direct effects

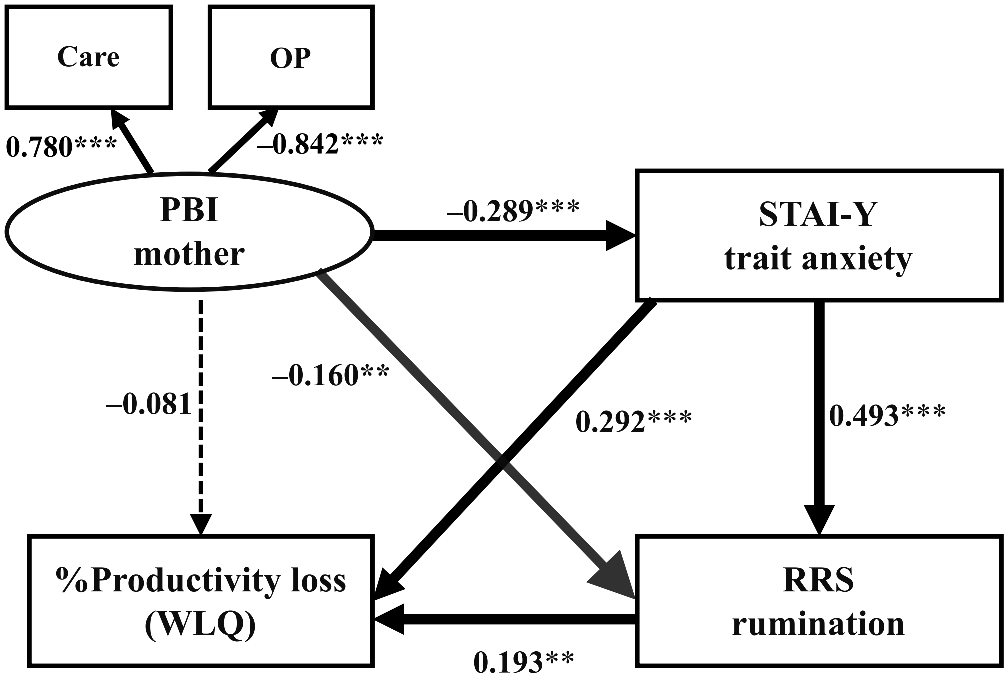

(B) Indirect effects

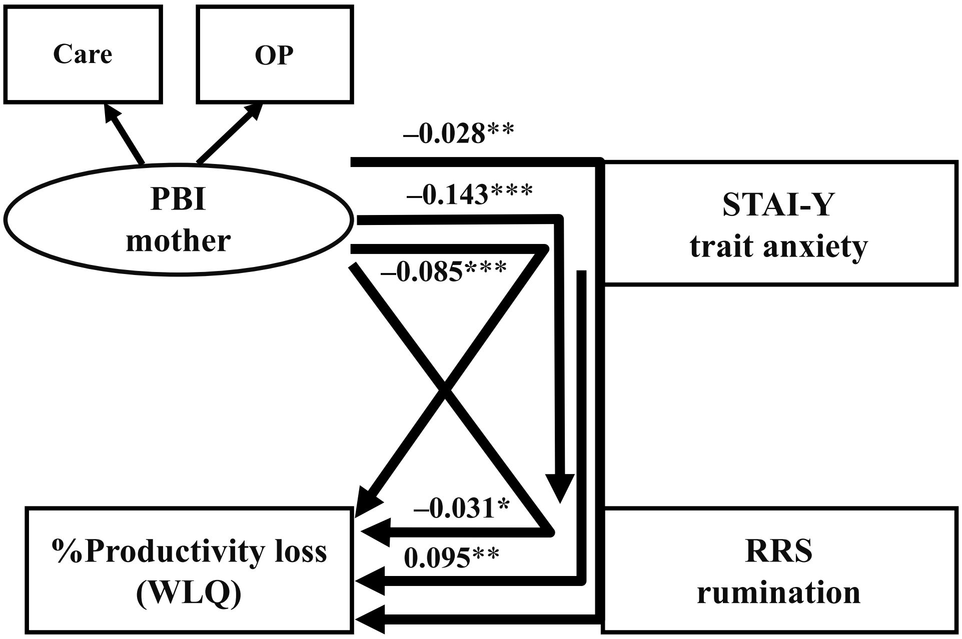

S1 Fig. 1. Results of the structural equation model with "PBI mother" as the latent variable, and care and overprotection (OP) of the PBI, trait anxiety (STAI-Y), depressive rumination (RRS), and %productivity loss (presenteeism) on the WLQ as the observed variables. The latent variable is shown as an oval, and the observed variables are shown as rectangles. Direct effects (A) and indirect effects (B) between the variables are shown. The numbers show the standardized path coefficients. *$p < 0.05$, **$p < 0.01$, ***$p < 0.001$

(A) Direct effects

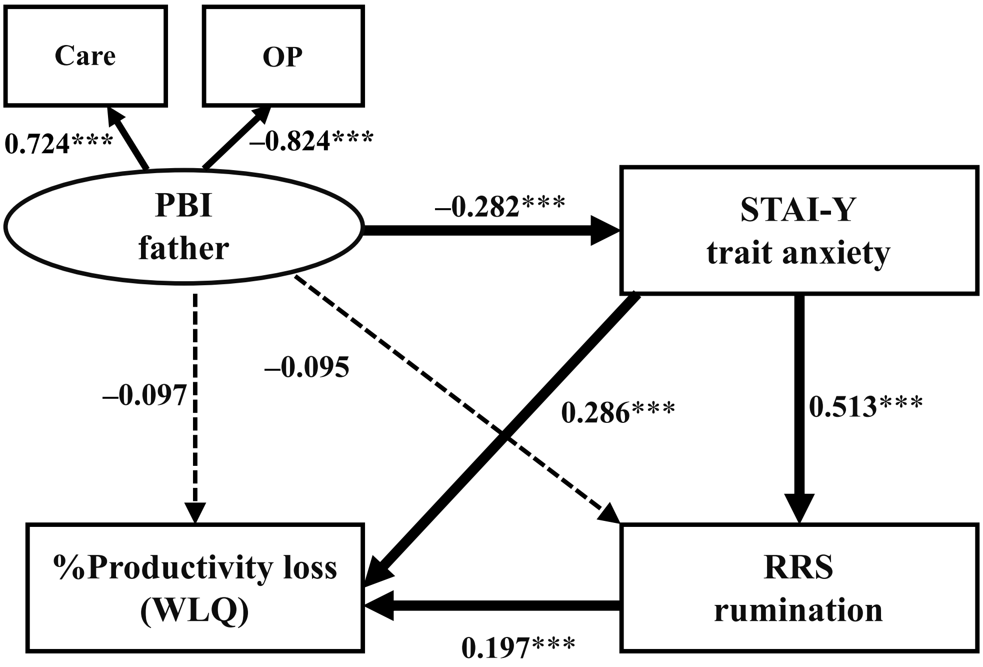

(B) Indirect effects

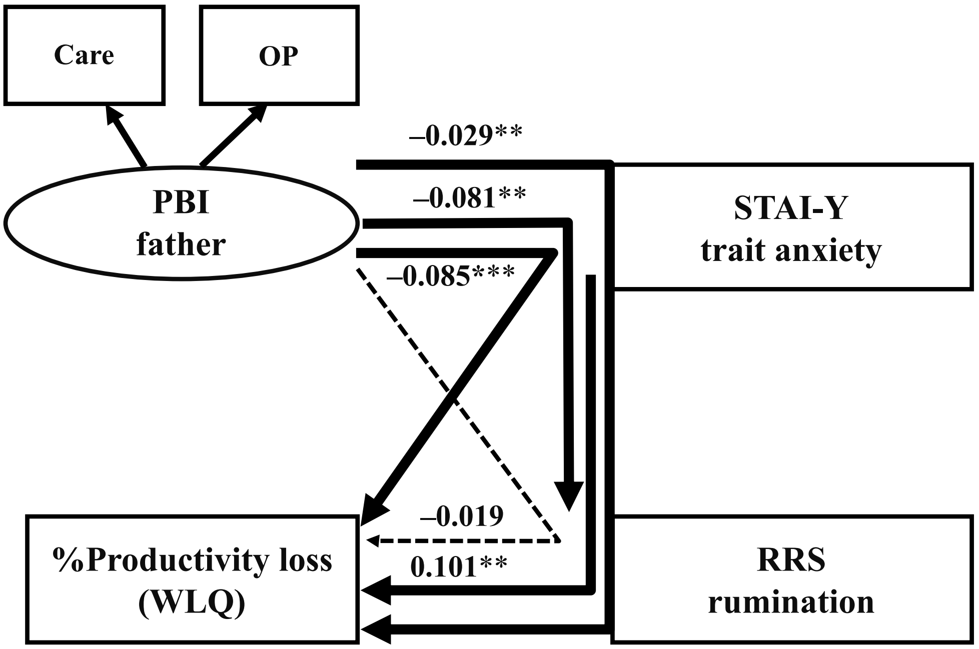

S1 Fig. 2. Results of the structural equation model with "PBI father" as the latent variable, and care and overprotection (OP) of the PBI, trait anxiety (STAI-Y), depressive rumination (RRS), and %productivity loss (presenteeism) on the WLQ as the observed variables. The latent variable is shown as an oval, and the observed variables are shown as rectangles. Direct effects (A) and indirect effects (B) between the variables are shown. The numbers show the standardized path coefficients. $^{**}p < 0.01$, $^{***}p < 0.001$
